# Improving medication safety and efficiency in hospital pharmacy through a pharmacist-led, low-code mobile application: a prospective study

**DOI:** 10.3389/fpubh.2026.1844863

**Published:** 2026-07-15

**Authors:** Bin Li, Xin Chen, Lijuan Xiong

**Affiliations:** Department of Pharmacy, The Second Affiliated Hospital of Shantou University Medical College, Shantou, Guangdong, China

**Keywords:** agile development, hospital pharmacy, low-code platforms, mobile inventory, patient safety

## Abstract

**Background:**

Traditional manual inventory management in hospital pharmacies is error-prone and inefficient, jeopardizing patient safety. Low-code platforms enable clinicians to build software, but their impact needs rigorous evaluation. To evaluate a pharmacist-led, low-code mobile inventory application’s impact on operational efficiency, accuracy, and labor in a hospital pharmacy.

**Methods:**

A prospective observational study was conducted over 12 months in a 1,460-bed tertiary hospital (formulary: 3,850 items). Pharmacists used a low-code platform to design, build, and iteratively refine a mobile inventory application through agile sprints. The application was deployed across twelve pharmacy subzones. Primary outcomes were inventory cycle time, error rate, stock variance, and labor allocation. Analysis included paired comparisons, multiple linear regression, discrete-event simulation for scalability projection, and sensitivity analysis.

**Results:**

Average weekly inventory cycle time decreased by 60.3%, from 14.6 ± 2.3 to 5.8 ± 1.1 h (*p* < 0.001). The inventory error rate decreased by 75.9%, from 8.7% ± 1.2 to 2.1% ± 0.7% (*p* < 0.001). Stock variance per SKU fell from 14.2 ± 3.6 to 3.7 ± 1.2 units. Pharmacist-led iterations (1.6/month) correlated with error reduction (R^2^ = 0.81, *p* < 0.001). Time on manual tasks decreased by 67.4%, reallocating 3.2 full-time equivalent (FTE) hours/week/pharmacist to clinical duties. Simulation projected error rates would stay below 3.0% with a 10% inventory increase.

**Conclusion:**

The pharmacist-led, low-code model was associated with marked improvements in pharmacy inventory management, showing substantial gains in efficiency, accuracy, and clinical time reallocation. It offers a scalable, cost-effective framework for healthcare digitization centered on domain expertise.

## 1 Introduction

Hospital pharmacy operations are critically dependent on the precise management of complex drug inventories to ensure patient safety and therapeutic efficacy. In the face of growing patient volumes and increasingly specialized formularies, traditional inventory systems, reliant on manual counting and paper-based logs, are fundamentally inadequate. These methods are intrinsically prone to human error, delayed reporting, and inefficient resource allocation, leading directly to stock discrepancies, medication shortages, and wastage, all of which compromise care quality and operational resilience (1, 2).

Consequently, the digitization of pharmacy workflows has emerged as an imperative. Evidence consistently demonstrates that digital interventions, from electronic prescription systems to automated dispensing, can significantly reduce errors, streamline processes, and improve patient outcomes (3–5). Mobile health technologies, in particular, offer potent advantages through real-time data capture and verification, enhancing traceability and clinical decision-making (6, 7). However, the successful adoption of such technologies is not guaranteed. Implementation success is heavily contingent on factors including institutional readiness, infrastructure, user training, and, critically, the system’s alignment with complex, dynamic clinical workflows (8–10). Off-the-shelf software often suffers from a rigidity that fails to accommodate the nuanced, zone-specific demands of a hospital pharmacy, leading to workarounds, low user engagement, and suboptimal returns on investment.

This gap between generic digital tools and context-specific operational needs highlights the potential of two convergent innovations: low-code development platforms and agile, domain-expert-led design. Low-code platforms democratize application development by enabling the creation of functional software through visual interfaces and pre-built components, drastically reducing the need for specialized programming skills (11). Seminal work has established low-code platforms as viable tools for business process automation and highlighted their application in healthcare information systems (12–14). When combined with agile methodologies, which emphasize iterative development and continuous user feedback, they offer a pathway to highly adaptable, fit-for-purpose solutions. The effectiveness of agile approaches in large-scale health information system implementation and usability-focused electronic health record design has been demonstrated (15, 16). Crucially, this paradigm empowers domain experts, the frontline clinicians who best understand workflow intricacies and safety requirements, to become direct contributors to the digital solution (17). This approach aligns with the broader finding that user engagement and perceived usefulness are paramount for the adoption of health information systems (18, 19).

In a hospital pharmacy, the domain expert is the pharmacist. Pharmacist-led development of digital tools represents a compelling strategy to bridge the persistent gap between clinical insight and technological implementation. Initial studies on digital pharmacy services, such as closed-loop management systems and telepharmacy, underscore the importance of workflow integration and staff involvement (20, 21). Furthermore, frameworks for interoperable digital medication records emphasize the need for technical standards that support, rather than constrain, local practice (22). Cross-cultural validation of agile health informatics approaches has been explored in comparative analyses across different healthcare systems (23). Despite this conceptual promise, there is a striking paucity of empirical, quantitative research evaluating the operational impact of a pharmacist-led, low-code development model for creating tailored mobile inventory applications within a large, complex hospital setting.

Therefore, this study aims to address this evidence gap. We present a prospective evaluation of a pharmacist-designed mobile inventory application built on a low-code platform in a 1,460-bed tertiary hospital. The primary objectives are: (1) to quantify the impact of this intervention on key operational metrics, including inventory error rates, processing time, and labor allocation; (2) to analyze the relationship between pharmacist-led iterative development and system performance; and (3) to model the scalability and robustness of the solution under increased operational load. By doing so, this research seeks to provide a validated, practical framework for agile and cost-effective digital transformation in hospital pharmacy operations.

## 2 Materials and methods

### 2.1 Study design, setting, and units of analysis

This study employed a prospective, observational design to evaluate the impact of a pharmacist-led mobile inventory application. The intervention was implemented and monitored over 12 months in the pharmacy department of a 1,460-bed tertiary care hospital with an active formulary of 3,850 drug items.

**Figure 1:**
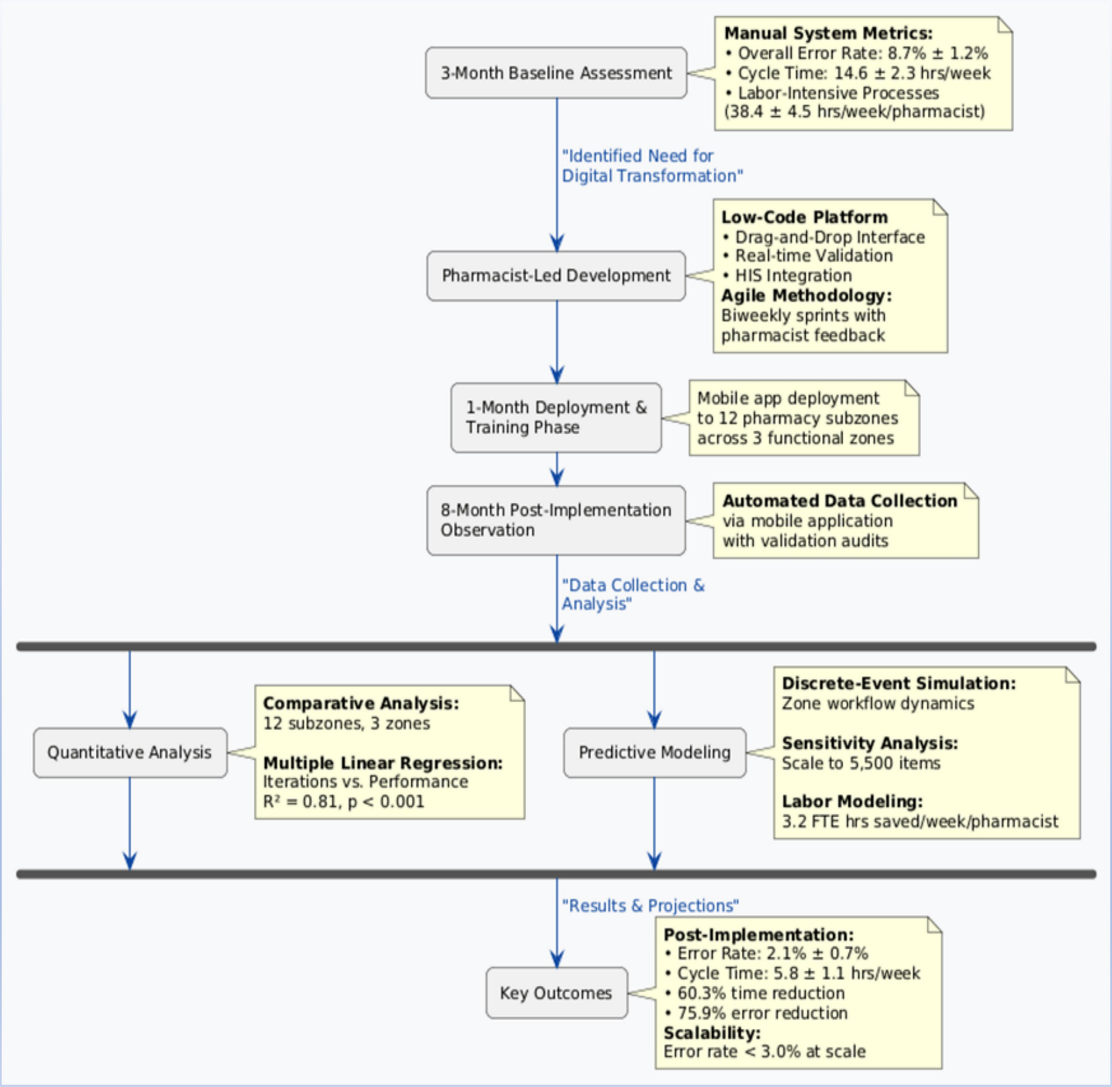
Implementation and evaluation roadmap for the pharmacist-led mobile inventory system.

To accurately capture the heterogeneity of hospital pharmacy workflows, operations were categorized into three primary functional zones: (1) Inpatient Services, (2) Outpatient Dispensary, and (3) Critical Care Services. These zones represent fundamentally different inventory demands, turnover rates, and clinical urgencies. For granular performance measurement and validation, data were collected from twelve distinct physical subzones nested within these three functional zones. The subzones comprised: Inpatient General Wards 1 & 2, Inpatient Oncology Ward, Outpatient Dispensaries A & B, Critical Care Units 1 & 2, a Specialty ICU Pharmacy, two Satellite Dispensaries, a Pediatric Ward Pharmacy, and the Emergency Pharmacy Unit. This two-tiered structure allowed for high-level modeling by functional zone and detailed performance reporting by physical location. The overall implementation and evaluation framework is summarized in Figure 1.

A 3-month pre-implementation phase established baseline metrics through manual time-motion studies and physical inventory audits. This was followed by a 1-month deployment and training period and an 8-month post-implementation observation phase.

Ethical approval was obtained from The Second Affiliated Hospital of Shantou University Medical College, Provincial People’s Hospital Medical Ethics Committee (Approval No. 2023-KY-0847). Written informed consent was obtained from all 22 participating pharmacists. No patient-identifiable data were accessed; all data were de-identified and stored on encrypted hospital servers. The study was conducted in accordance with the Declaration of Helsinki.

### 2.2 Intervention: pharmacist-led low-code application development

The core intervention was the development and deployment of a custom mobile inventory application using Mendix Studio Pro 9.24.2 LTS, a commercial low-code development platform. The platform enabled drag-and-drop interface design, real-time data validation, and integration with the existing Hospital Information System (HIS) via HL7 FHIR R4 APIs. The application was deployed on-premise within the hospital’s private cloud, utilizing a PostgreSQL 14 database managed by hospital IT. Authentication and access control were implemented via OAuth 2.0 integrated with Hospital Active Directory, requiring multi-factor authentication for all users. All data transmissions were encrypted using TLS 1.3. The system maintained complete audit logs of all inventory transactions, including user identity, timestamp, and action type, to support fraud detection and drug diversion monitoring.

The development process was explicitly pharmacist-led. A core team of staff pharmacists, in collaboration with a single IT facilitator, translated operational requirements directly into application logic. Development followed agile principles and was conducted in bi-weekly sprints. This iterative cycle allowed for continuous refinement of features, such as barcode scanning interfaces, low-stock alert thresholds, and cold-chain tracking modules, based on direct pharmacist feedback and observed workflow bottlenecks. This approach ensured the tool was tailored to specific needs within each functional zone, including protocols for controlled substances and high-cost biologics.

### 2.3 Data collection and variables

Data collection spanned a total 12-month period, comprising sequential phases: a 3-month pre-implementation baseline, a 1-month deployment and training phase, and an 8-month post-implementation observation phase. Pre-implementation metrics were established via manual time-motion studies and physical inventory audits. Post-implementation data were captured primarily through automated logs generated by the mobile application. To validate the accuracy of the digital measurements against a gold standard, we conducted parallel manual audits on a randomly selected 10% of SKUs per subzone per week (*n* = 468 total validation audits). Inter-rater reliability between manual and digital counts was assessed using intraclass correlation coefficients (ICC) and Bland–Altman analysis (presented in Supplementary Figure S1A). The mean difference between methods was −0.12 units (95% limits of agreement: −1.48 to 1.24), indicating minimal systematic bias. Key operational variables included: Inventory Cycle Time (weekly pharmacist-hours for full count/reconciliation), Inventory Error Rate (percentage discrepancy between recorded and physical stock), Stock Variance (mean absolute unit discrepancy per SKU), Labor Allocation (weekly hours on manual inventory tasks), and Application Iterations (monthly count of pharmacist-led modifications). Aggregated metrics for the three primary functional zones, which served as inputs for subsequent modeling, are detailed in Supplementary Table S1 (baseline) and Supplementary Table S2 (post-implementation).

### 2.4 Statistical analysis and modeling

A hybrid analytical framework was employed to evaluate the intervention’s impact and underlying mechanisms.

#### 2.4.1 Primary comparative analysis

Paired comparisons of pre- and post-implementation metrics (cycle time, error rate, labor allocation, SKU variance) were performed for each of the twelve subzones using paired t-tests (or Wilcoxon signed-rank tests for variables that violated normality assumptions). Normality of the paired differences was assessed using the Shapiro–Wilk test (*p* > 0.05 for all primary outcomes, indicating no significant deviation from normality). Results for the subzones are presented as mean ± standard deviation, with percentage reductions calculated to demonstrate effect size. Aggregated comparisons at the level of the three primary functional zones are also reported.

#### 2.4.2 Regression modeling

Multiple linear regression was used to analyze the relationship between pharmacist engagement, measured as the number of application iterations per month (independent variable), and improvements in system performance (dependent variables: reduction in error rate and reduction in SKU variance). Model fit was assessed using the R^2^ statistic.

#### 2.4.3 Discrete-event simulation (DES)

A simulation model was built to represent the inventory workflow dynamics of the three primary functional zones. The model was calibrated using baseline data from Supplementary Table S1 and validated against observed post-implementation outcomes from Supplementary Table S2. Key model assumptions (Poisson-distributed demand with *λ* = 145/h, lognormal service times with mean 12.5 s, first-order Markov error propagation with transition probabilities p₀₁ = 0.087 and p₁₁ = 0.45) and comprehensive sensitivity analysis are fully detailed in the Methods in Supplementary material.

#### 2.4.4 Sensitivity and scalability analysis

Using the validated DES model, sensitivity analyses were conducted to project system robustness. The model simulated increased operational loads by scaling the number of managed SKUs from 4,500 to 5,500 items to predict key outcome metrics (error rate, cycle time) under stress and confirm scalability.

The complete mathematical formulation of the discrete-event simulation, regression, and projection models is provided in the Methods in Supplementary material.

## 3 Results

### 3.1 Operational efficiency improvements

**Figure 2:**
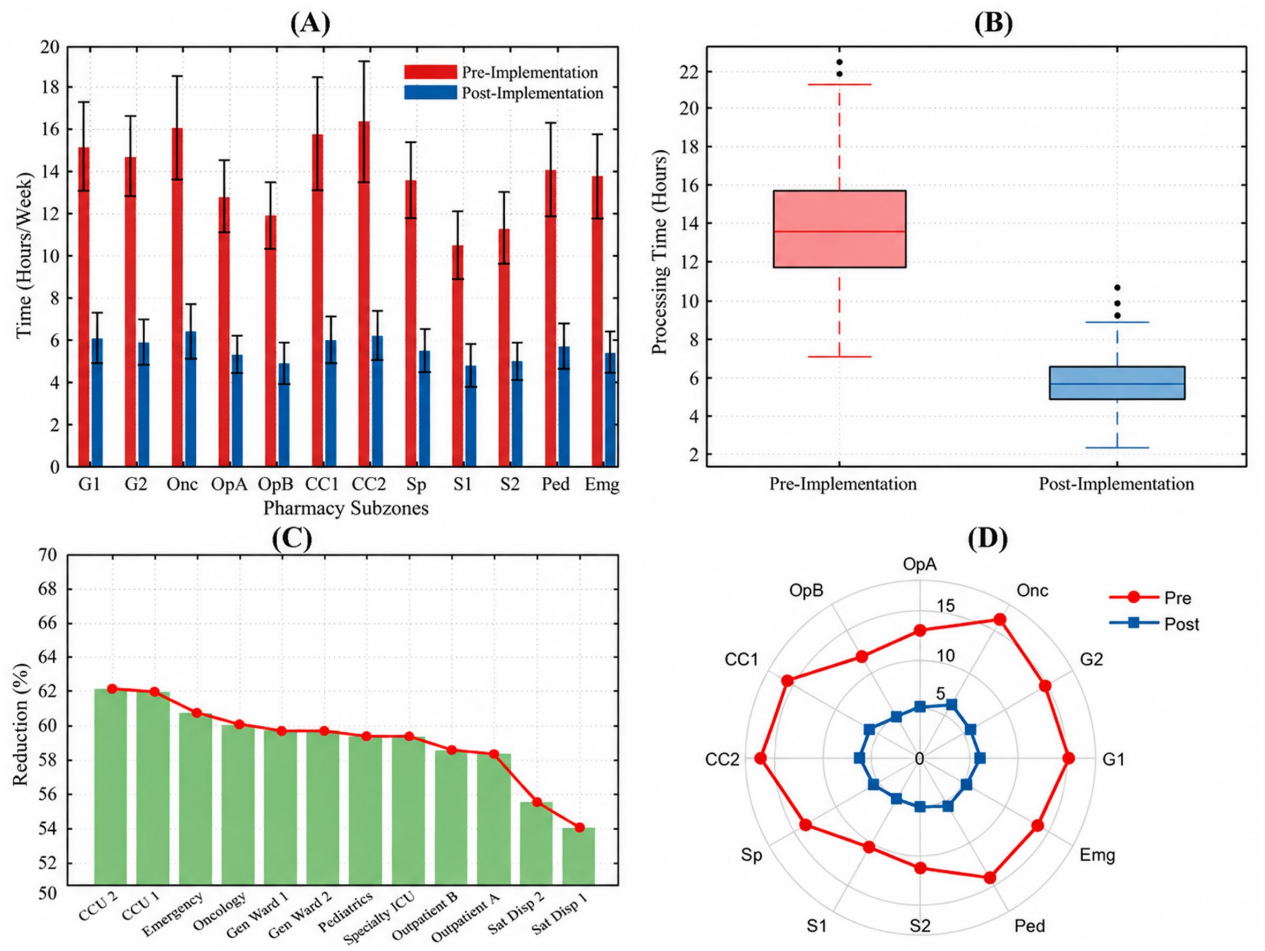
Operational efficiency gains following mobile inventory application deployment. **(A)** Comparison of mean weekly inventory cycle times (hours) pre- and post-implementation across subzones. **(B)** Box plots showing the distribution and reduction in cycle time variability. **(C)** Percentage reduction in cycle time per subzone, ranked by efficiency gain. **(D)** Radial diagram illustrating the systemic contraction of operational load profiles post-implementation.

Following the deployment of the pharmacist-led mobile inventory application, inventory processing time was significantly reduced across all pharmacy zones. The average weekly inventory cycle time decreased from 14.6 ± 2.3 h to 5.8 ± 1.1 h, corresponding to a 60.3% reduction (*p* < 0.001). Figure 2 illustrates the compression of workflow variability and the consistent efficiency gains across all subzones.

Subzone-level analysis confirmed substantial and consistent improvements (Table 1). Pre-implementation cycle times ranged from 10.5 ± 1.6 to 16.4 ± 2.9 h per week. Post-implementation times decreased to a range of 4.8 ± 1.0 to 6.4 ± 1.3 h, representing percentage reductions between 54.3 and 64.1%. The greatest absolute time saving (10.2 h/week) and largest percentage gain (62.2%) were both observed in Critical Care Unit 2.

**Table 1:** Inventory cycle times across pharmacy subzones.

Pharmacy subzone	Pre-implementation (hours/week)	Post-implementation (hours/week)	Reduction (%)
Inpatient General Ward 1	15.2 ± 2.1	6.1 ± 1.2	59.9
Inpatient General Ward 2	14.7 ± 1.9	5.9 ± 1.1	59.9
Inpatient Oncology Ward	16.1 ± 2.5	6.4 ± 1.3	60.2
Outpatient Dispensary A	12.8 ± 1.7	5.3 ± 0.9	58.6
Outpatient Dispensary B	11.9 ± 1.6	4.9 ± 1.0	58.8
Critical Care Unit 1	15.8 ± 2.7	6.0 ± 1.1	62.0
Critical Care Unit 2	16.4 ± 2.9	6.2 ± 1.2	62.2
Specialty Pharmacy – ICU	13.6 ± 1.8	5.5 ± 1.0	59.6
Satellite Dispensary 1	10.5 ± 1.6	4.8 ± 1.0	54.3
Satellite Dispensary 2	11.3 ± 1.7	5.0 ± 0.9	55.8
Pediatric Ward Pharmacy	14.1 ± 2.2	5.7 ± 1.1	59.6
Emergency Pharmacy Unit	13.8 ± 2.0	5.4 ± 1.0	60.9

Data are mean ± SD. Source: Automated application logs and on-site verification.

### 3.2 Inventory accuracy and error reduction

Following implementation of the mobile application, a pronounced improvement in inventory record accuracy was observed. The overall inventory error rate decreased from 8.7% ± 1.2 to 2.1% ± 0.7%, representing a 75.9% reduction (*p* < 0.001). Concurrently, the mean stock variance per SKU decreased from 14.2 ± 3.6 units to 3.7 ± 1.2 units.

The improvement was consistent across all twelve subzones (Table 2). Pre-implementation error rates ranged from 7.3 to 9.6%, falling to a post-implementation range of 1.8 to 2.4%. Stock variance decreased from a pre-implementation range of 12.5 ± 2.6 to 16.1 ± 3.9 units per SKU to a post-implementation range of 3.0 ± 0.9 to 4.2 ± 1.2 units.

**Table 2:** Inventory accuracy metrics across pharmacy subzones.

Pharmacy subzone	Pre error rate (%)	Post error rate (%)	Pre SKU variance (units)	Post SKU variance (units)
Inpatient General Ward 1	8.9 ± 1.1	2.2 ± 0.6	14.3 ± 3.5	3.8 ± 1.1
Inpatient General Ward 2	9.2 ± 1.2	2.3 ± 0.7	15.0 ± 3.8	4.0 ± 1.2
Inpatient Oncology Ward	9.6 ± 1.3	2.4 ± 0.7	15.5 ± 3.9	4.2 ± 1.2
Outpatient Dispensary A	7.8 ± 1.0	1.9 ± 0.5	13.1 ± 2.8	3.2 ± 0.9
Outpatient Dispensary B	7.5 ± 0.9	1.8 ± 0.5	12.8 ± 2.6	3.0 ± 0.9
Critical Care Unit 1	9.4 ± 1.3	2.3 ± 0.7	15.2 ± 3.9	4.1 ± 1.2
Critical Care Unit 2	9.1 ± 1.2	2.2 ± 0.6	14.8 ± 3.6	3.9 ± 1.1
Specialty Pharmacy – ICU	8.3 ± 1.0	2.0 ± 0.6	13.5 ± 3.1	3.5 ± 1.0
Satellite Dispensary 1	7.6 ± 0.9	1.9 ± 0.5	12.5 ± 2.6	3.1 ± 0.9
Satellite Dispensary 2	7.3 ± 0.9	1.8 ± 0.5	12.8 ± 2.7	3.0 ± 0.9
Pediatric Ward Pharmacy	8.7 ± 1.1	2.1 ± 0.6	14.0 ± 3.4	3.7 ± 1.1
Emergency Pharmacy Unit	8.5 ± 1.0	2.1 ± 0.6	13.8 ± 3.2	3.6 ± 1.0

Data are mean ± SD. SKU Variance = mean absolute unit discrepancy per SKU. Source: Automated application logs and manual audits.

Regression analysis revealed a strong, significant correlation between the frequency of pharmacist-led application iterations per month and the reduction in error rates (R^2^ = 0.81, *p* < 0.001; *β* = −0.824, SE = 0.118, 95% CI: −1.057 to −0.591). After controlling for months post-implementation as a covariate, the independent effect of iterations remained significant (*β* = −0.824, SE = 0.118, 95% CI: −1.057 to −0.591, *p* < 0.001).

The discrete-event simulation model, validated against the post-implementation outcomes, projected that error rates would remain below 3.0% even under a simulated 10% increase in inventory load.

**Figure 3:**
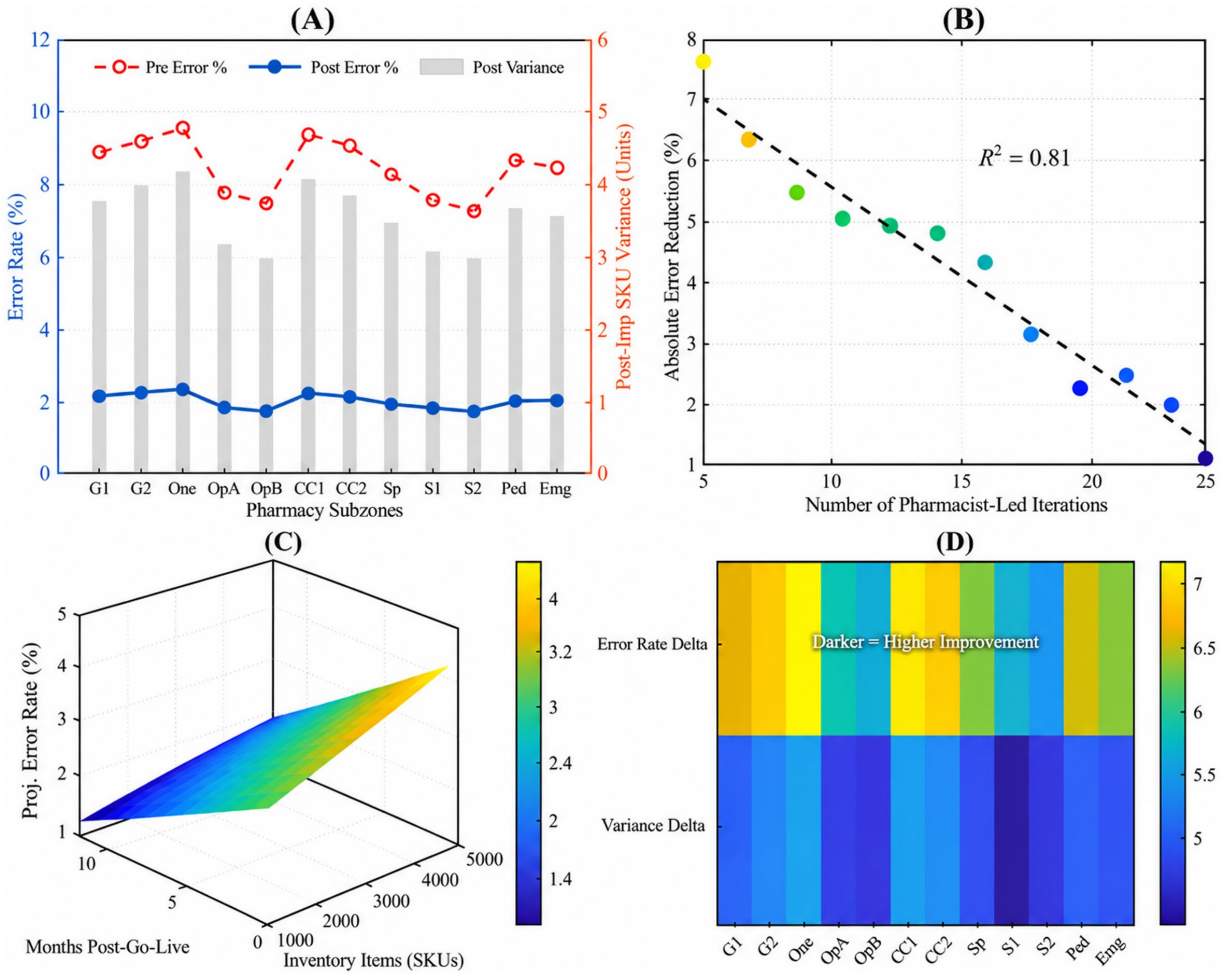
Inventory accuracy improvements and system robustness. **(A)** Dual-axis plot showing the reduction in error rates (line) and residual SKU variance (bars) post-intervention. **(B)** Linear correlation (R^2^ = 0.81, *p* < 0.001) between the number of pharmacist-led agile iterations and the reduction in error rates. **(C)** 3D sensitivity analysis surface projecting stable error rates (<3.0%) under scenarios of increasing inventory scale and time. **(D)** Heatmap of performance improvement deltas across pharmacy subzones.

Figure 3 visualizes the reduction in error rates and residual variance, and the correlation between pharmacist engagement and accuracy gains.

### 3.3 Resource allocation and pharmacist engagement

The intervention significantly altered pharmacist labor allocation. A total of 22 pharmacists participated in the study across the 12 subzones (Table 3). The time devoted to manual inventory tasks decreased from an average of 38.4 ± 4.5 h to 12.5 h (range: 11.2–13.5) per pharmacist per week, a reduction of 67.4%. This equated to a reallocation of 3.2 FTE hours per pharmacist per week to clinical activities. The breakdown of labor savings across all subzones is shown in Table 3.

**Table 3:** Pharmacist labor allocation and reallocation across subzones.

Pharmacy subzone	N (Pharmacists)	Pre-implementation (hours/week)	Post-implementation (hours/week)	Reduction (%)	Redeployed to clinical tasks (hours/week)
Inpatient General Ward 1	2	39.0 ± 3.8	12.8 ± 2.1	67.2	26.2
Inpatient General Ward 2	2	39.2 ± 3.9	13.0 ± 2.2	66.8	26.2
Inpatient Oncology Ward	2	40.1 ± 4.2	13.5 ± 2.3	66.3	26.6
Outpatient Dispensary A	2	36.5 ± 4.2	11.5 ± 1.9	68.5	25.0
Outpatient Dispensary B	2	36.0 ± 3.7	11.2 ± 1.7	68.9	24.8
Critical Care Unit 1	2	39.7 ± 4.5	13.2 ± 2.3	66.8	26.5
Critical Care Unit 2	2	39.5 ± 4.1	12.9 ± 2.1	67.4	26.6
Specialty Pharmacy – ICU	2	37.8 ± 3.6	11.8 ± 2.0	68.8	26.0
Satellite Dispensary 1	1	35.8 ± 3.2	11.2 ± 1.7	68.7	24.6
Satellite Dispensary 2	1	36.3 ± 3.5	11.4 ± 1.8	68.5	24.9
Pediatric Ward Pharmacy	2	38.6 ± 3.9	12.5 ± 2.1	67.6	26.1
Emergency Pharmacy Unit	2	40.2 ± 4.5	13.3 ± 2.2	66.9	26.9
Total	22	—	—	—	—

Data are mean ± SD. Source: Personnel work logs and automated application tracking. N = number of participating pharmacists per subzone. Total *N* = 22 pharmacist.

Pharmacists actively engaged in refining the application, with an average of 1.6 application iterations per month logged across the study period. Statistical validation of the data, including agreement analysis between manual and digital counts and the probability shift in cycle times, is presented in Supplementary Figure S1. The correlation between key outcomes and the projected annual clinical capacity gain is shown in Supplementary Figure S2.

## 4 Discussion

This prospective study found that a pharmacist-led, low-code development model was associated with substantial improvements in hospital pharmacy inventory management. Following the intervention, we observed a 60.3% reduction in weekly inventory cycle time, a 75.9% reduction in error rates, and the reallocation of 3.2 FTE hours per week per pharmacist from administrative tasks to clinical activities. While these improvements coincided with the intervention, we cannot rule out alternative explanations including Hawthorne effects, concurrent process changes, seasonal variation, or staff experience accumulation over time. Therefore, findings should be interpreted as associations rather than causal effects.

### 4.1 The central role of domain expertise in agile digital health development

The core innovation of this study was placing pharmacists, the primary end-users and workflow experts, at the center of the application development process. This approach was associated with direct translation of clinical knowledge into application logic via a low-code platform, potentially circumventing a significant barrier in health informatic: the misalignment between externally developed software and complex, nuanced clinical workflows (9, 10). The significant correlation between the frequency of pharmacist-led iterations (averaging 1.6 per month) and the reduction in error rates (R^2^ = 0.81, *p* < 0.001) provides quantitative evidence for a key theoretical principle: continuous, context-aware adaptation by end-users is critical for system effectiveness and reliability (18).

Our findings align with and extend recent literature on digital transformation in healthcare settings. The agile, iterative process mirrors the adaptive digital solutions highlighted as crucial for managing public health emergencies (17) and supports the systematic, criteria-driven approach to optimizing pharmacy resource allocation (24). The success of this model likely stems from its ability to leverage the “installed base” of pharmacist expertise, akin to how pre-existing digital infrastructure facilitates information exchange among practitioners (25), and translate it directly into functional software.

### 4.2 Operational and patient safety implications

The observed improvements in inventory accuracy (error rate: 8.7 to 2.1%; stock variance: 14.2 to 3.7 units/SKU) suggest direct and meaningful implications for patient safety, though causality cannot be inferred from this study design. Inaccurate inventory is a root cause of medication errors, including treatment delays and incorrect dosing. Our post-intervention error rate of 2.1% is comparable to benchmarks set by advanced, automated closed-loop medication systems (1, 4), yet was achieved through a flexible, mobile-first tool rather than fixed, capital-intensive automation. The system’s robustness in high-acuity environments like critical care units is particularly noteworthy, as these areas are most vulnerable to safety incidents stemming from stock discrepancies (26).

Furthermore, the intervention transformed the pharmacist’s workflow. By automating manual counting and data transcription, it reversed the common trend where digital tools add to clinical burden (9). The reallocation of nearly 70% of inventory-related labor (saving 3.2 FTE hours/pharmacist/week) enabled a shift toward higher-value activities like patient counseling, medication verification, and therapeutic optimization, core components of pharmaceutical care linked to improved outcomes (7, 21). This aligns with global findings that digitization, when properly aligned with workflow, can enhance both efficiency and clinical performance (5, 27).

### 4.3 Scalability, economic viability, and a model for digital transformation

The scalability of this model is one of its most compelling features. The discrete-event simulation predicted that error rates would remain below 3.0% even with a 10% increase in inventory load, demonstrating robust scalability. This projection is supported by the high agreement between digital and manual audit methods and the significant compression of workflow variability.

Economically, the model presents a viable pathway for digitization. The modest initial investment in platform subscription and training was offset by substantial labor cost savings, suggesting potential cost-effectiveness. This challenges the perception that meaningful digital transformation requires prohibitively significant capital expenditure (8) and offers a pragmatic model for resource-constrained settings. The use of a low-code platform also future-proofs the investment. By empowering pharmacists to modify the application, the system can evolve with changing formularies and protocols. For broader institutional integration, future iterations could incorporate cybersecurity considerations (28) and interoperability standards (22) to enhance data exchange and security.

### 4.4 Limitations and future directions

This study has several limitations. First, the single-arm, pre-post design without a control group precludes causal inference; improvements may reflect Hawthorne effects, concurrent process changes, seasonal variation, or staff experience accumulation. Findings should be interpreted as associations only. Second, the correlation between iteration frequency and error reduction (R^2^ = 0.81) does not imply causation; iteration count may proxy for engagement or time, residual confounding may exist, and mediation analysis was beyond scope. Third, the single-center design in a large Chinese tertiary hospital limits generalizability to other settings. Fourth, systematic measurement bias is possible; although validation audits showed high reliability (ICC = 0.94, Supplementary Figure S1), residual bias cannot be excluded. Fifth, inventory accuracy is an upstream proxy for safety; we did not measure adverse drug events or clinical harm. Additionally, our error rate definition does not distinguish errors by clinical severity (e.g., missing a life-saving drug vs. miscounting a low-risk vitamin). Future studies should stratify errors by potential clinical impact. Sixth, the pharmacist-led model requires clinician engagement and digital literacy, which vary across institutions. Seventh, we did not assess human factors or cognitive load; future studies should incorporate standardized ergonomic and usability assessments (e.g., NASA-TLX, SUS). Eighth, the intervention was not integrated with broader medication safety ecosystems (CPOE, CDSS, BCMA); full system-level integration is needed for closed-loop safety. Ninth, the percentage reductions in cycle time (54.3 to 64.1%) and error rates (73.8 to 76.3%) were remarkably consistent across the 12 functionally distinct subzones. This uniformity may reflect the standardized nature of the intervention (same application logic, identical training protocol, and unified agile development process applied across all subzones), rather than true equivalence of workflow complexity. Alternatively, the Hawthorne effect may have produced similar improvements regardless of baseline differences. Readers should interpret this consistency with caution, as greater variability might be expected in routine practice.

Future research should focus on multi-center randomized controlled trials to establish causal efficacy across different hospital types and cultures. Longitudinal studies are needed to evaluate the sustainability of both the performance gains and the agile development culture. Furthermore, research should explore the integration of these mobile low-code applications with broader hospital interoperability standards like FHIR, and assess cybersecurity aspects specific to pharmacy applications (22, 29).

## 5 Conclusion

This study provides robust evidence that a pharmacist-led, low-code development model was associated with a highly effective and efficient strategy for hospital pharmacy digitization. Following implementation, we observed a 60.3% reduction in weekly inventory processing time and a 75.9% decrease in inventory error rates, while reallocating 3.2 full-time equivalent hours per pharmacist per week from administrative counting to clinical verification and patient safety activities. Predictive modeling confirmed the approach’s scalability, projecting sustained error rates below 3.0% even with increased inventory loads. By moving beyond implementing off-the-shelf technology to co-creating agile digital tools with frontline experts, this model demonstrates a fundamental strategy for building resilient, adaptive, and human-centered digital health infrastructure. It offers a replicable, cost-effective framework that enhances both operational performance and, ultimately, patient safety.

## Data Availability

The original contributions presented in the study are included in the article/Supplementary material, further inquiries can be directed to the corresponding author.
