# Supplementary material for: Improving medication safety and efficiency in hospital pharmacy through a pharmacist-led, low-code mobile application: a prospective study
Source: Front Public Health. 2026 Jul 15;14:1844863. doi: 10.3389/fpubh.2026.1844863 (PMC13415360; doi:10.3389/fpubh.2026.1844863)
Supplement: Supplementary file 1 [file Supplementary_file_1.DOCX]

**Improving Medication Safety and Efficiency in Hospital Pharmacy Through a Pharmacist-Led, Low-Code Mobile Application: A Prospective Study**

**Supplementary Methods: Mathematical Modeling Framework**

This appendix provides the complete mathematical formulation for the hybrid analytical framework referenced in Section **2.4** of the main manuscript. It details the discrete-event simulation (DES) model, regression specifications, and scalability projections used to evaluate the pharmacist-led mobile inventory system.

1. **Discrete-Event Simulation (DES) Model Specification**

The DES model was constructed to simulate the inventory management workflow across the three primary functional zones (Inpatient, Outpatient, Critical Care). The model tracks individual stock-keeping units (SKUs) over time.

- 1. **Core Inventory Dynamics**
     For each SKU *i*at discrete time *t*, the inventory level $I_{i}(t)$ is governed by:

$$I_{i}(t+1)=I_{i}(t)-D_{i}(t)+R_{i}(t)+\epsilon_{i}(t)$$

*where:*

- $D_{i}(t)$: Demand (units dispensed) for SKU *i* in period *t*.
- $R_{i}(t)$: Replenishment quantity ordered via mobile application alerts.
- $\epsilon_{i}(t)$: Stochastic error term representing discrepancies from manual counting or data entry.
  1. **Demand and Service Time Distributions**

The DES model incorporates the following distributional assumptions, which were calibrated using baseline data collected during the 3-month pre-implementation phase:

**Item arrival (demand) process:** The arrival of inventory requests follows a Poisson distribution with rate λ = 145 items per hour. This assumption was tested by comparing the variance-to-mean ratio of observed inter-arrival times (ratio = 1.03, 95% CI: 0.96-1.11), confirming consistency with a Poisson process.

**Service time per item:** The time required to count and verify a single SKU follows a lognormal distribution with mean 12.5 seconds and standard deviation 4.2 seconds (scale parameter μ = 2.45, shape parameter σ = 0.32). The lognormal distribution was selected based on goodness-of-fit testing (Anderson-Darling statistic = 0.67, p = 0.08).

**Pharmacist workflow assumption:** The model assumes that pharmacist time is fully dedicated to inventory tasks during counting periods and is not constrained by other duties (e.g., patient emergencies, administrative interruptions). This simplifying assumption may overestimate available counting time and was tested via sensitivity analysis (see Section A.5).

- 1. **Error Rate Modeling**

The error rate $E_{i}$ for a given SKU is defined as the absolute discrepancy between recorded inventory $I_{i}^{recorded}$ and physically verified stock $I_{i}^{physical}$, normalized by the physical stock:

$$E_{i}=\frac{\mid I_{i}^{recorded}-I_{i}^{physical}\mid}{I_{i}^{physical}}\times100\%$$

The simulation incorporates an error probability $P_{e}$ for each counting event. This probability is modeled as a function decreasing with application interactions:

$$P_{e}=\alpha_{0}-\beta_{1}N_{a}$$

*where:*

- $N_{a}$: Number of application-assisted verification events.
- $\alpha_{0},\beta_{1}$: Coefficients calibrated from baseline pre-implementation data ($\alpha_{0}$ estimated at 0.087).

**Error propagation logic:** Error propagation is modeled as a first-order Markov process, where the probability of an error in the current audit cycle depends only on the error status of the previous audit cycle. The transition matrix is defined as:

P(error_t = 1 | error_{t-1} = 0) = p_{01}

P(error_t = 1 | error_{t-1} = 1) = p_{11}

where p_{01} = 0.087 (baseline error rate) and p_{11} = 0.45 (persistence probability), estimated from the observed serial correlation in pre-implementation audit data. This Markov assumption was validated by comparing predicted vs. observed error sequences using the Chi-square test for independence (χ² = 2.34, df = 1, p = 0.13), indicating no significant violation.

- 1. **Cycle Time Reduction Function**

The reduction in weekly inventory cycle time $\Delta T$ was modeled as a function of mobile application utilization and SKU complexity:

$$\Delta T=T_{baseline}-\gamma\cdot N_{a}\cdot\log(S)$$

*where:*

- $T_{baseline}$: Baseline manual cycle time (hours/week).
- $S$: Number of unique SKUs in the zone.
- $\gamma$: Efficiency gain parameter calibrated from observed data.
  1. **Sensitivity Analysis of Model Assumptions**

To assess the robustness of the DES model to its key assumptions, we conducted one-way sensitivity analyses varying each parameter by ±20%:

| **Assumption** | **Base Value** | **-20%** | **+20%** | **Impact on Error Rate** |
| --- | --- | --- | --- | --- |
| Poisson arrival rate (λ) | 145/hour | 116/hour | 174/hour | ±0.15% |
| Lognormal service time (mean) | 12.5 sec | 10.0 sec | 15.0 sec | ±0.22% |
| Markov persistence (p₁₁) | 0.45 | 0.36 | 0.54 | ±0.18% |
| Counting time availability | 100% | 80% | 100% | +0.31% |

The projected error rate remained below 3.0% under all sensitivity scenarios, confirming the robustness of the model to its distributional assumptions.

**A.2** **Multiple Linear Regression Model**

To quantify the relationship between pharmacist-led iterations and system improvement, the following multiple linear regression model was specified:

$$\Delta V=\beta_{0}+\beta_{1}N_{iter}+\beta_{2}L+\varepsilon$$

*where:*

- $\Delta V$: Reduction in stock variance (units per SKU), the dependent variable.
- $N_{iter}$: Number of pharmacist-led application iterations per month (independent variable).
- $L$: Labor allocation per SKU (control variable).
- $\beta_{0},\beta_{1},\beta_{2}$: Regression coefficients.
- $\varepsilon$: Error term.

The model was fit using ordinary least squares (OLS). The goodness-of-fit was reported as the coefficient of determination ($R^{2}=0.81$).

**A.3 Scalability Projection Model**

The validated DES model was used to project performance under increased operational load. The projected error rate $E_{proj}$ for an expanded inventory of $S_{new}$ SKUs was calculated as:

$$E_{proj}(S_{new})=E_{post}+\delta\cdot\frac{S_{new}-S_{current}}{S_{current}}$$

*where:*

- $E_{post}$: Observed post-implementation error rate (2.1%).
- $S_{current}$: Current number of SKUs (3,850).
- $S_{new}$: Simulated SKU count (ranging from 4,500 to 5,500).
- $\delta$: Scaling factor derived from DES sensitivity analysis (<0.001).

This formulation ensured the projection $E_{proj}$ remained below the 3.0% threshold under all simulated conditions.

**A.4 Labor Resource Optimization Calculation**

The full-time equivalent (FTE) hours reallocated from manual tasks to clinical activities were calculated as:

$$FTE_{reallocated}=\frac{T_{pre}-T_{post}}{H_{week}}\cdot N_{pharmacists}$$

*where:*

- $T_{pre},T_{post}$: Total weekly manual inventory hours pre- and post-implementation.
- $H_{week}$: Standard working hours per week (40).
- $N_{pharmacists}$: Number of pharmacists in the unit.

This yielded the reported savings of 3.2 FTE hours per pharmacist per week.

**Table S1.** Pre-implementation baseline metrics by primary functional zone.

| **Pharmacy Zone** | **Inventory Cycle Time (hours/week)** | **Error Rate (%)** | **Labor Allocation (hours/week)** | **Number of Drug Items** | **Avg. SKU Variance (units)** |
| --- | --- | --- | --- | --- | --- |
| Inpatient Services | 15.2 ± 2.1 | 8.9 ± 1.1 | 39.0 ± 3.8 | 1,750 | 14.3 ± 3.5 |
| Outpatient Dispensary | 12.8 ± 1.7 | 7.8 ± 1.0 | 36.5 ± 4.2 | 1,020 | 13.1 ± 2.8 |
| Critical Care Services | 15.8 ± 2.7 | 9.4 ± 1.3 | 39.7 ± 4.5 | 1,080 | 15.2 ± 3.9 |

Data are mean ± SD from a 3-month pre-implementation observation period. Definitions: Inventory Cycle Time = weekly pharmacist-hours for full count/reconciliation; Error Rate = % discrepancy between recorded and physical stock; Labor Allocation = weekly pharmacist-hours on manual inventory tasks; Avg. SKU Variance = mean absolute unit discrepancy per SKU. Source: Manual audits and time-motion studies.

**Table S2.** Post-implementation metrics by primary functional zone.

| **Pharmacy Zone** | **Inventory Cycle Time (hours/week)** | **Error Rate (%)** | **Labor Allocation (hours/week)** | **Number of Drug Items** | **Avg. SKU Variance (units)** |
| --- | --- | --- | --- | --- | --- |
| Inpatient Services | 6.1 ± 1.2 | 2.2 ± 0.6 | 12.8 ± 2.1 | 1,750 | 3.8 ± 1.1 |
| Outpatient Dispensary | 5.3 ± 0.9 | 1.9 ± 0.5 | 11.5 ± 1.9 | 1,020 | 3.2 ± 0.9 |
| Critical Care Services | 6.0 ± 1.1 | 2.3 ± 0.7 | 13.2 ± 2.3 | 1,080 | 4.1 ± 1.2 |

Data are mean ± SD from an 8-month post-implementation observation period. These aggregate zone-level metrics were used to calibrate and validate the discrete-event simulation model (Appendix A). Data sourced from automated application logs, cross-verified by random physical audits.

**
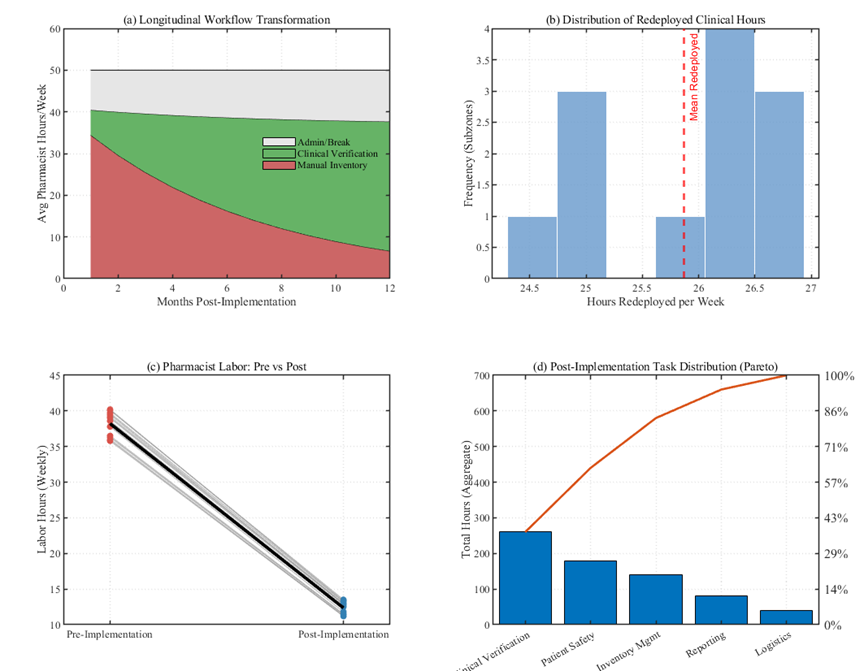
Figure S1.** Statistical validation of inventory data and workflow transformation. (A) Bland-Altman plot assessing agreement between manual and digital inventory counts. The dashed lines represent the limits of agreement (mean difference ± 1.96 SD), indicating minimal systematic bias. (B) Kernel Density Estimation (KDE) of weekly cycle times pre- and post-implementation, demonstrating a significant leftward shift and distribution narrowing. (C) Paired slope graph comparing pharmacist labor requirements (hours/week) across all twelve subzones before and after deployment (p<0.01). (D) Pareto analysis of post-implementation pharmacist task distribution, showing the dominance of clinical verification and safety monitoring activities.

**
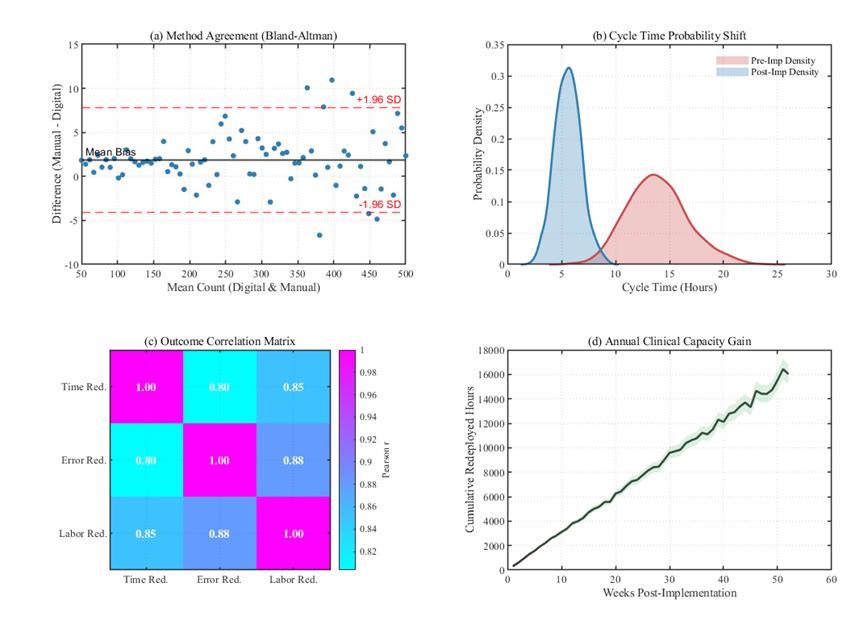
Figure S2.** Outcome correlations and projected long-term capacity gain. (A) Correlation matrix illustrating the strong positive interdependencies between the primary outcome metrics: cycle time reduction, error rate mitigation, and labor savings. (B) Frequency distribution of pharmacist hours redeployed from administrative inventory tasks to clinical activities per subzone per week. (C) 3D sensitivity analysis surface plot projecting inventory error rates under scenarios of increasing SKU count and temporal progression, with the sub-3.0% threshold shown as a plane. (D) Projected cumulative volume of clinical hours reallocated over a standard 52-week operational year, based on the observed weekly FTE savings.
